# Cognitive Assessment of Geriatric Patients in Primary Care Settings

**DOI:** 10.7759/cureus.10443

**Published:** 2020-09-14

**Authors:** Eukesh Ranjit, Amit Sapra, Priyanka Bhandari, Christine E Albers, Mohitkumar S Ajmeri

**Affiliations:** 1 Department of Family and Community Medicine, Southern Illinois University School of Medicine, Springfield, USA

**Keywords:** primary care, geriatrics, cognitive assessment, memory issues, dementia, mild cognitive impairment, alzheimer's, vascular dementia, clinical assessment, private practice anesthesia

## Abstract

Cognitive decline is a common issue seen in older adults in the primary care setting. Assessment of cognitive decline in primary care includes a detailed history, physical examination, labs, imaging, and a formal cognitive assessment. Various tools are available for cognitive assessments. However, a short screening tool is more practical for cognitive evaluation. A decline in cognition should be correlated with the broader clinical picture, and a detailed cognitive assessment should be performed. This article focuses on some of the cognitive assessment tools used in clinical settings to assess cognition.

## Introduction and background

Primary care is often the first medical setting where patients with cognitive symptoms present for consultation. A decline in cognition and dementia are commonly seen in older adults. Dementia is a condition characterized by a decline in one or more cognitive domains that are significant enough to interfere with daily life [1]. The Diagnostic and Statistical Manual of Mental Disorders, Fifth Edition (DSM-5) has recognized and defined the following domains for evaluating a neurocognitive disorder: complex attention, executive function, learning and memory, language, and perceptual-motor and social cognition.

Clinical assessment of all six domains requires a considerable amount of time, which is not always available in a primary care setting. Also, patients with cognitive decline may present with multiple comorbidities and often with issues with communication (hearing, vision, and comprehension). Various clinical assessment tools have been developed to assess cognitive decline, which, together with other clinical findings, aid in the assessment of cognition.

## Review

Epidemiology and screening

Dementia is primarily a disease of older adults. It is one of the leading causes of mortality in older adults. Various neurocognitive diseases can lead to dementia. The most common among them are Alzheimer’s (60-80%), vascular dementia (5-10%), Lewy body dementia (5-10%), frontotemporal dementia (5-10%), and others (Parkinson’s, Huntington’s, etc.) [2]. Also, in a number of cases, the etiology might be mixed with more than one condition present.

Alzheimer’s disease, one of the leading causes of dementia, accounts for 5.8% of deaths in people over the age of 65 years and 9.2% of deaths in those over the age of 85 years in the United States (US); it is the sixth leading cause of death in the US [3,4]. As of 2014, 1.6% of the US population had Alzheimer’s and related dementias, and the number is projected to grow to 3.3% in 2060 [5]. Despite its prevalence, there is no consensus on standard screening for the condition. The United States Preventive Service Task Force (USPSTF) has graded screening for cognitive impairment in older adults as grade “I,” meaning that evidence is insufficient to determine the benefits and harms of screening. History, physical examination, lab studies, imaging, and cognitive assessment tools form the core of assessment for dementia in primary care settings.

Before assessing cognition, it is essential to rule out delirium as delirium can often be confused with dementia. Confusion Assessment Method (CAM) can be used to assess delirium. Lab tests, including complete blood count (CBC), basic metabolic panel (BMP), thyroid-stimulating hormone (TSH), and vitamin B12 can be used as baseline labs to rule out conditions like anemia, electrolyte abnormalities, and hypothyroidism, which can influence cognition [6]. Imaging modalities of the head, including CT scan, can be used to rule out reversible conditions like normal pressure hydrocephalus and to assess the extent of atrophy of the brain. Depression and anxiety can affect cognition and hence should be ruled out prior to cognitive assessment.

Various clinical tools have been developed for cognitive assessment. It is worth noting that cognitive assessment methods do not diagnose dementia and are just screening tools to assess cognition [6]. Some of the common tools are described below.

Mini-Cog^©^

Mini-Cog^©^ is a short cognitive assessment test that can be performed in a primary care setting. The test consists of two parts: the ability to recall three words and a clock drawing. Out of a total score of 5, 1 point each is granted for each word recalled, and either 0 or 2 points for clock drawing. Because of its simplicity, it is easy to administer in an outpatient setting where time is essential. A positive Mini-Cog should be followed up with a more elaborate cognitive assessment test. According to a meta-analysis, the sensitivity of the diagnostic accuracy of Mini-Cog was 91% and specificity was 86% [7].

Mini-Mental Status Exam (MMSE)

The Mini-Mental Status Exam (MMSE) is one of the most widely used tests for cognitive assessment and one of the most frequently studied dementia screening tests [7]. It consists of a total of 20 questions with a maximum MMSE score of 30 points. Although it is a screening tool, scores have been calibrated to suggest the extent of cognitive impairment (Table 1) [8]. For patients with established dementia, MMSE is used to classify the severity of disease into mild, moderate, and severe types. According to a systemic review and meta-analysis, the sensitivity and specificity of MMSE for dementia detection were 81% and 89%, respectively [7].

**Table 1:** Classification of cognitive impairment with MMSE scores MMSE: Mini-Mental Status Exam

Score	Severity
24-30	No cognitive impairment
18-23	Mild cognitive impairment
0-17	Severe cognitive impairment

Montreal Cognitive Assessment (MoCA)

The Montreal Cognitive Assessment (MoCA) is one of the screening tools for cognitive disorders. This tool evaluates visuospatial/executive, naming, memory, attention, language, abstraction, delayed recall, and orientation. The total score in the test is 30. Per the developer, the results are intended to be interpreted by healthcare professionals with expertise in cognitive fields. The tool is proprietary [9] and requires training and certification to be used [10].

Saint Louis University Mental Status (SLUMS) exam

The Saint Louis University Mental Status (SLUMS) exam has 11 items with a total score of 30. Scoring is based on the education levels of the patient (Table 2) [11].

**Table 2:** SLUMS exam score interpretation SLUMS: Saint Louis University Mental Status

	High-school education	Less than high-school education
Normal	27-30	25-30
Mild neurocognitive disorder	21-26	20-24
Dementia	1-20	1-19

A pilot study has indicated that SLUMS might be better at detecting mild neurocognitive disorders [12]. The test is non-proprietary and free for clinicians to use. SLUMS is limited in use and has less published data associated with it compared to MMSE [13].

Rowland Universal Dementia Assessment Scale (RUDAS)

The Rowland Universal Dementia Assessment Scale (RUDAS) was developed in Australia. It is a six-item tool with a maximum score of 30. The test was designed to be used with culturally and linguistically diverse communities [14]. The sensitivity and specificity of the tool have been reported to be 89% and 98%, respectively. A review of linguistically and culturally diverse populations outside Australia found the sensitivity and specificity mean of the tool to be 80.9% and 76.1%, respectively [14].

## Conclusions

Primary care physicians are often the first contact for patients with cognitive issues. A combination of detailed history, examination, lab, imaging modalities, and utilization of cognitive assessment tools are needed for a proper cognitive assessment. A number of cognitive assessment tools are available to assess patients. Depending on the clinical setting and provider’s familiarity with the assessment tool, patients with cognitive issues should be screened, if warranted.
